# Biosensors for oral cancer detection: a scoping review

**DOI:** 10.1186/s13000-026-01796-6

**Published:** 2026-06-22

**Authors:** Achmad Syawqie, Gita Dwi Jiwanda Sovira, Amaliya-Amaliya, Solachuddin Jauhari Arief Ichwan

**Affiliations:** 1 Department of Oral Biology, Faculty of Dentistry, Universitas Padjadjaran, Bandung, Indonesia; 2 Dentistry Programme PAPRSB Institute of Health Sciences, Universiti Brunei Darussalam, Bandar Seri Begawan, Brunei

## Abstract

This scoping review summarizes recent advances in biosensor technologies for the early, non-invasive detection of oral cancer, with a focus on salivary biomarkers and point-of-care platforms. Five databases (PubMed, Scopus, Web of Science, ProQuest, and EBSCO) were searched for studies published from 2019 to 2025. After duplicate removal and screening, nine eligible original studies were charted. The included platforms were primarily electrochemical, optical, and transistor-based biosensors targeting biomarkers such as interleukin-8 (IL-8), cytokeratin fragment 21.1 (Cyfra 21.1), cancerous inhibitor of PP2A (CIP2A/P90), and high-risk HPV genotypes. Reported limits of detection were frequently in the femtomolar range, although reporting of assay time, sample volume, and clinical diagnostic accuracy was inconsistent. Overall, biosensors show strong analytical potential for oral cancer screening; however, translation to routine clinical use will require standardized analytical validation, careful control of pre-analytical saliva variables, and well-designed multicenter clinical studies reporting sensitivity and specificity against appropriate reference standards.

## Introduction

Oral cancer is one of the most prevalent malignancies worldwide, posing a significant public health burden [1]. It is considered the sixth most common cancer in the world and primarily includes malignancies of the lips and oral cavity; cancers of the oropharyngeal region (e.g., the base of tongue) are often reported separately but share several risk factors. According to the Global Cancer Observatory (GLOBOCAN) and the International Agency for Research on Cancer (IARC), more than 0.3 million new cases of oral and lip cancer and over 0.1 million deaths were reported worldwide in 2020 [1, 2]. In several countries, oral cancer is one of the most common types of cancer among men and ranks third among women. The disease is particularly prevalent in regions with high tobacco and alcohol consumption, such as South Asia, Eastern Europe, and parts of Latin America. Oral squamous cell carcinoma (OSCC) is the most common and severe type of the disease and generally occurs in the lateral edge of the tongue, the mouth floor, buccal mucosa, and gingiva [3]. Furthermore, this type of cancer accounts for approximately 90% of oral cancer diagnostic cases. Sex differences are consistently observed, with higher incidence in men than women, which may reflect differences in exposure to high-risk habits. Geographical distribution varies substantially, and the regions with the highest incidence rates are in South and Southeast Asia. Sri Lanka, Bangladesh, Pakistan, and India are known as the countries with the highest rates of oral cancer for men, where 25% of all cancer cases are diagnosed as oral cancer [4]. Due to population aging and continued exposure to multiple risk factors, it is estimated that the number of oral cancer patients will reach 856,000 by 2035 [1].

Numerous risk factors contribute to the development of oral cancer, with variations observed based on geographical prevalence. In developed countries, smoking and excessive alcohol consumption are identified as the primary causes, accounting for approximately 75% of oral cancer cases [5]. Conversely, in developing countries, in addition to tobacco smoking, the practice of chewing areca nut and betel quid, with or without tobacco, is regarded as one of the most significant risk factors. Furthermore, a diet high in nitrosamines, such as salted fish, has been associated with an increased risk of oral cancer. Infections caused by high-risk strains of the human papillomavirus (HR-HPV), particularly HPV-16 and HPV-18, have been strongly linked to cancers arising in the posterior oral cavity and oropharyngeal region, while the Epstein-Barr virus (EBV) is also being investigated as a potential contributor to the rising incidence of oral cancer [6].

Accurately determining the stage of oral cancer is crucial, as early detection significantly improves patient outcomes.6 When malignancy is identified at stage T1, the reported 5-year survival rate can reach approximately 90%; however, this figure may decline to around 20% in advanced stages such as T3–T4. At stage T4, oral cancer progresses to a high-grade metastatic phase, spreading primarily through the lymphatic system to other organs. During metastasis, cancer cells travel via the lymphatic pathways, with the cervical lymph nodes being the primary site of oral cancer metastasis. Additionally, the lungs serve as a significant secondary target for the migration of oral cancer cells. Consequently, early detection and preventive measures, particularly through screening programs targeting high-risk populations with precancerous lesions, represent an effective approach to controlling the disease at its initial stage (T1) and enhancing treatment efficacy [5–7].

One of the major challenges in oral cancer diagnosis is the absence of a screening method that is non-invasive, rapid, highly sensitive, accurate, and cost-effective. Various techniques have been developed for the early detection of oral cancer neoplasia, including toluidine blue staining, oral cytology (brush biopsy), fluorescence spectroscopy, imaging, chemiluminescent illumination, and biopsy [8]. However, reports indicate that the diagnostic process for oral cancer can take up to six months. Currently, the most reliable diagnostic approach remains oral tissue biopsy followed by histopathological examination, Polymerase Chain Reaction (PCR), Enzyme-Linked Immunosorbent Assay (ELISA), and mass spectrometry. Despite their effectiveness, these methods are generally costly, invasive, time-consuming, and require specialized equipment and trained professionals. Additionally, sample analysis—including blood, serum, and tissue—often necessitates extensive pre-treatment. Given the increasing prevalence of cancer, further research is essential to identify novel biomarkers with high specificity and sensitivity while developing affordable and non-invasive diagnostic techniques [9–11].

Biomarkers are quantifiable biological indicators composed of DNA, RNA, or proteins that offer valuable insights into the body's health status or disease progression. These molecules are detectable in various bodily fluids, including saliva, blood, serum, and plasma, as well as in tissue samples obtained from different organs [10, 11]. The evaluation of salivary biomarkers presents a promising, non-invasive, safe, and cost-effective alternative for the clinical diagnosis of oral cancer, making it a potential tool for early detection and disease monitoring. Whole saliva is a complex biological fluid produced by the combined secretion of three major salivary glands: the sublingual, parotid, and submandibular glands, along with numerous minor salivary glands. In addition to salivary gland secretions, saliva contains various other components, including sputum, nasal secretions, blood from oral wounds or bleeding, exfoliated epithelial cells, and food debris. Furthermore, saliva serves as a host to a diverse range of microorganisms, such as bacteria, viruses, and fungi [11–13].

Biochemically, saliva is a clear fluid with an average protein concentration ranging from 1.5 to 2 mg/mL. It holds significant potential for early disease detection due to the presence of a diverse array of biomolecules. In recent years, numerous studies have explored the use of saliva in diagnosing various cancers, including lung, breast, oral, and pancreatic cancer, highlighting its potential as a diagnostic tool for a wide range of diseases [13, 14]. Moreover, compared to blood and serum, saliva presents a less complex biological matrix, as the presence of numerous macromolecules in blood and serum can interfere with diagnostic accuracy. Whole saliva has the potential to be a precise and dependable tool for the early-stage diagnosis of oral cancer due to its direct interaction with the oral cavity. Currently, researchers have identified over 100 potential biomarkers in saliva, highlighting its growing significance as a non-invasive diagnostic tool for various diseases, including oral cancer [12, 14, 15].

Biosensors are analytical devices capable of detecting specific biomolecules with high sensitivity and in a short time. In the context of oral cancer, biomarkers such as interleukin-8 (IL-8) have been identified as critical indicators that can be detected using electrode-based biosensors and nanotechnology materials. The use of biosensors allows for biomarker measurements in biological samples such as saliva, enabling non-invasive early detection [14].

Electrode-based biosensor technology has advanced rapidly, particularly with the integration of materials such as graphene oxide, carbon nanotubes, and metal oxide nanoparticles. For instance, nano-ceria-based biosensors have been proven to enhance stability, sensitivity, and selectivity in detecting the IL-8 biomarker [4, 16]. With these advances, oral cancer detection can be performed more quickly and accurately, facilitating earlier medical intervention. However, analytical performance can differ substantially between purified targets (e.g., buffer systems) and complex clinical matrices such as whole saliva, emphasizing the importance of validation in clinically relevant samples for point-of-care use. Given the rising incidence of oral cancer and the limitations of conventional methods, biosensors offer an innovative solution that is more efficient, economical, and accessible for early disease detection [16, 17]. Therefore, further research into the development and application of biosensors for oral cancer is essential in improving diagnostic strategies and patient treatment outcomes.

## Material and methods

### Study design

The study design employed in this study is a scoping review, conducted in accordance with the PRISMA 2020 guidelines for study selection. Findings were synthesized narratively, and no meta-analysis was performed because of heterogeneity in target biomarkers, biosensor platforms, and outcome reporting.

### Literature search and eligibility criteria

This scoping review conducted a literature search of articles using the MeSH (Medical Subject Headings) terminology (Supplementary Table). We searched PubMed, Scopus, Web of Science, Proquest, and EBSCO from 2019 to 2025 to identify publications reporting biosensor-based detection of oral cancer–related biomarkers (including salivary, blood, serum, plasma, tissue, or cell-based samples). Keywords used included "biosensors" OR "biomarkers" OR “salivary biomarker” OR “blood biomarker” OR “oral biosensor” OR “blood biosensor” AND "oral cancer," "oral carcinoma," and "Oral Squamous Cell Carcinoma." Studies were eligible if they were original articles (including experimental studies and clinical reports) reporting biosensor development and/or analytical validation for oral cancer or oral potentially malignant disorder biomarkers. Review articles were used for background context but were not charted as primary studies. Data were synthesized qualitatively to present a comprehensive understanding of biosensor-based approaches for oral cancer detection.

### Prisma flowchart

**Fig. 1:**
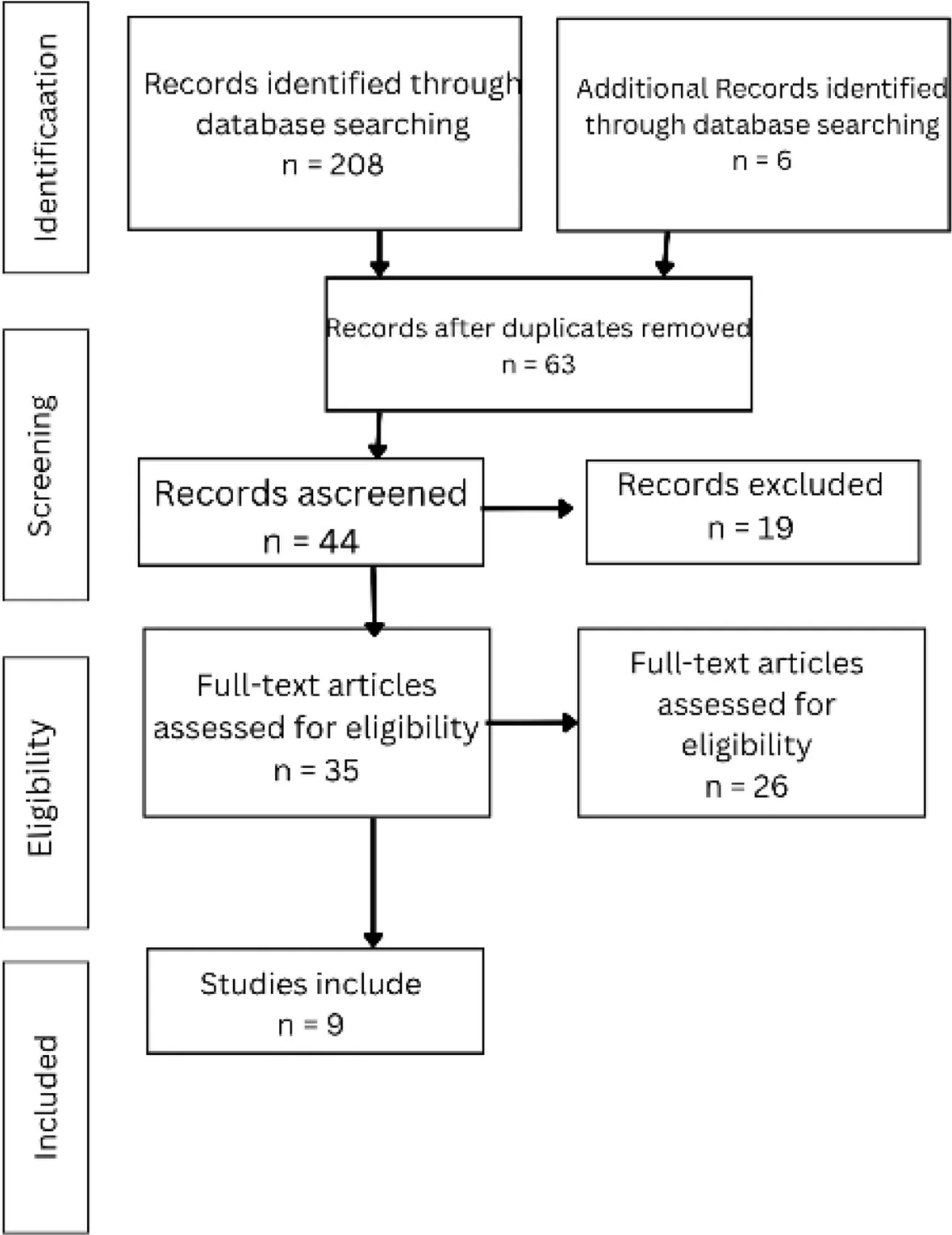
PRISMA flowchart

Article selection followed an iterative team approach using a PRISMA flowchart. A total of 214 records were identified (208 through database searching and 6 additional records). After removal of duplicates, 63 unique records remained. Of these, 19 records were excluded as clearly irrelevant to biosensor-based oral cancer detection, and 44 records were screened further. Full-text articles were assessed for eligibility (*n* = 35), and 26 articles were excluded at the full-text stage for reasons such as not focusing on oral cancer, not using a biosensor-based detection approach, being a review/editorial, or not reporting sufficient analytical information. Ultimately, 9 original studies were included and charted (Fig. 1).

### Quality appraisal and reporting framework

Because the included articles comprised a mixture of analytical biosensor development studies and studies using clinical specimens, study appraisal focused on two complementary dimensions. For studies reporting diagnostic performance using clinical samples, risk of bias and applicability were considered using a QUADAS-2 domain structure (patient selection, index test, reference standard, and flow/timing) based on information available in the reports. For primarily analytical or proof-of-concept studies, we extracted and compared analytical validation parameters (e.g., limit of detection, dynamic/linear range, and matrix type) and noted whether validation was performed in purified/buffer systems, spiked matrices, or unprocessed clinical samples.

### Data extraction

Filtering out unrelated publications and organizing the remaining information into a table, which includes the article title, author(s), publication year, country of origin, study objective, research design, sample size/participants (when available), and key findings. Where reported, analytical performance characteristics (e.g., limit of detection and dynamic range) and validation matrices were also extracted to support cross-study comparison (Table 1).

**Table 1:** Data charting

No	Article Title	Author’s Name	Year	Type of Research	Purpose	Result
1	High sensitivity CIP2A detection for oral cancer using a rapid transistor-based biosensor module	Minghan Xian, et al	2022	Experimental	This article aims to develop and evaluate a highly sensitive saliva-based biosensor for detecting breast cancer biomarkers, HER2 and CA15-3, providing a non-invasive, rapid, and user-friendly diagnostic method	This study demonstrates that the developed saliva-based biosensor has an extremely low detection limit (as low as 1 fg/ml) and high sensitivity, surpassing conventional ELISA methods, and can serve as an efficient tool for early, non-invasive breast cancer detection
2	A Multianalyte Electrochemical Genosensor for the Detection of High-Risk HPV Genotypes in Oral and Cervical Cancers	Thanyarat Chaibun, et al	2022	Experimental	This article aims to develop a multianalyte electrochemical DNA biosensor for detecting high-risk HPV genotypes 16 and 18 in both oral and cervical cancer samples, providing a rapid, sensitive, and non-invasive diagnostic tool	The study concludes that the developed biosensor demonstrates high specificity and sensitivity, with detection limits as low as 22 fM for HPV-16 and 20 fM for HPV-18, making it a promising alternative for clinical HPV detection
3	An electrochemical immunosensor based on a nano-ceria integrated microfluidic chip for interleukin-8 biomarker detectionUse-Associated Lung Injury	Hema Bhardwaj, et al	2025	Experimental	This article aims to develop and evaluate a nano-ceria-integrated electrochemical immunosensor for the highly sensitive and selective detection of the IL-8 biomarker in saliva, facilitating early and non-invasive diagnosis of oral cancer	The developed electrochemical immunosensor demonstrated high sensitivity, selectivity, and stability, detecting IL-8 at very low concentrations (0.0001 ng/mL) in saliva samples, making it a promising tool for early-stage oral cancer detection
4	Expanding salivary biomarker detection by creating a 3 synthetic neuraminic acid sensor via chimeragenesis	Samuel J. Verzino, et al	2024	experimental research	This article aims to create a synthetic neuraminic acid sensor using chimeragenesis to expand salivary biomarker detection, providing a novel platform for early and non-invasive disease diagnosis	The study successfully developed a bacterial strain expressing a chimeric transcription factor (Sphnx) capable of detecting Neu5Ac, proving the feasibility of a WCB platform for potential clinical screening applications
5	Functionalized optical fiber ball-shaped biosensor for label-free, low-limit detection of IL-8 protein	Gyunel Rashidova, et al	2024	experimental research	The article aims to develop and validate an optical fiber biosensor for the highly sensitive and selective detection of IL-8 protein in saliva, offering a rapid and non-invasive diagnostic tool for disease monitoring	The study successfully demonstrated that the functionalized optical fiber biosensor could detect IL-8 at extremely low concentrations (as low as 0.91 fM), proving its high sensitivity, selectivity, and potential application in clinical diagnostics
6	High sensitivity saliva-based biosensor in detection of breast cancer biomarkers: HER2 and CA15-3	Hsiao-Hsuan Wan, et al	2024	Experimental study	To analyze various nanomaterial biosensors used for detecting salivary biomarkers in oral cancer diagnosis	The article is identified different nanomaterial biosensors with significant potential for early oral cancer detection, highlighting their advantages and detection capabilities
7	MoS2-ZnO Nanocomposite Mediated Immunosensor for Non-Invasive Electrochemical Detection of IL8 Oral Tumor Biomarker	Cittrarasu Vetrivel, et al	2023	Experimental study	The article aims to develop and evaluate a highly sensitive and selective electrochemical immunosensor using a MoS₂-ZnO nanocomposite to detect IL-8 in saliva, providing a rapid and cost-effective diagnostic tool for early-stage oral cancer detection	The developed immunosensor demonstrated excellent sensitivity, specificity, and stability, detecting IL-8 at ultra-low concentrations (as low as 11.6 fM), proving its potential as a reliable and efficient tool for non-invasive early diagnosis of oral cancer
8	Non-invasive bioassay of Cytokeratin Fragment 21.1 (Cyfra 21.1) protein in human saliva samples using immunoreaction method: An efficient platform for early-stage diagnosis of oral cancer based on biomedicine	Mohsen Jafari, et al	2020	Experimental research	The article aims to design and validate an efficient immunosensor for detecting Cyfra 21.1 in unprocessed human saliva samples, providing a rapid, sensitive, and cost-effective diagnostic platform for early-stage oral cancer detection	The developed immunosensor successfully detected Cyfra 21.1 in saliva with high sensitivity and specificity, demonstrating its potential as a reliable, non-invasive, and user-friendly tool for early diagnosis and monitoring of oral cancer
9	Sensitive Detection of Oral Leukoplakia: Analyzing P90 Biomarkers in Saliva and Tissue	Hsiao-Hsuan Wan, et al	2024	Experimental research	The article aims to introduce a high-sensitivity biosensor for detecting the P90 (CIP2A) protein in human saliva and tissue samples, providing a cost-effective and accurate diagnostic tool for identifying oral leukoplakia and its potential progression to oral cancer	The developed biosensor successfully detected P90 protein with a detection limit five orders lower than commercial ELISA kits, demonstrating its effectiveness in distinguishing oral leukoplakia samples and its potential for non-invasive early oral cancer screening

## Discussion

This scoping review investigated the current landscape of biosensor technologies for the early, non-invasive detection of oral squamous cell carcinoma (OSCC) and related oral potentially malignant disorders, with an emphasis on salivary biomarkers. The included studies were categorized based on their target biomarkers and sensing modalities, offering a structured overview of current innovations. Importantly, most included reports represent early-stage analytical or prototype development, and only a subset used unprocessed clinical specimens; therefore, conclusions about clinical diagnostic performance must be interpreted cautiously.

Traditional diagnostic techniques, such as histopathology, imaging (MRI, CT scans), and molecular testing (PCR, ELISA), are often expensive, time-consuming, and invasive [5]. Biosensors offer a promising alternative by providing rapid, cost-effective, and non-invasive detection methods [1]. Their advantages include high analytical sensitivity, the ability to analyze biomarkers in bodily fluids (such as saliva), real-time monitoring, and potential point-of-care applicability. Nevertheless, clinical usefulness depends not only on analytical sensitivity but also on reproducible performance in real-world samples and on clearly reported diagnostic accuracy metrics [3].

### Use of biosensors in oral cancer detection

Biosensors offer significant advantages in detecting oral cancer by providing a non-invasive, highly sensitive, and rapid diagnostic approach. Compared to traditional techniques such as biopsies, imaging, and histopathological examinations, biosensors may enable more frequent monitoring using saliva or tissue samples, thereby reducing patient discomfort. Across the included studies, several platforms achieved ultra-low analytical detection limits that can surpass conventional methods like ELISA; however, key reporting elements (e.g., assay time, sample volume, and precision) were not consistently provided, which limits direct comparability. In addition, the matrix used for validation varied across studies (buffer systems, cell lysates, spiked samples, and unprocessed saliva), and these differences can substantially affect performance in point-of-care settings [3, 18, 19].

### Mechanism of biosensors for oral cancer detection

The biosensors described in the reviewed studies employ various detection mechanisms, including electrochemical, optical, transistor-based, and metamaterial-based approaches. Electrochemical biosensors utilize nanomaterial-modified electrodes to enhance signal transduction and improve analytical sensitivity, enabling quantification of cancer-related molecules in saliva. Optical fiber biosensors function by detecting changes in light properties upon interaction with target proteins, enabling label-free analysis. Transistor-based biosensors use functionalized surfaces (often antibody-modified) to capture specific biomarkers and convert molecular binding events into measurable electrical signals. Additionally, flexible terahertz metamaterial biosensors have been proposed for real-time monitoring of cancer cell growth and migration, providing complementary insight into tumor progression [20–23].

To improve transparency and cross-study interpretability, Table 2 summarizes analytical performance metrics and the validation context reported for each included study. Where information was not reported (NR), it is highlighted to emphasize current gaps in reporting. Standardized reporting of limit of detection/quantification, dynamic range, assay time, sample volume, and precision—together with clear documentation of the biological matrix used—would strengthen future reviews and accelerate translation.

**Table 2:** Summary of analytical performance metrics and validation context reported across the included studies

Study (Author, year)	Target analyte	Platform/modality	Matrix tested (as reported)	LOD/LOQ (as reported)	Linear/Dynamic range	Assay time	Sample volume	Clinical diagnostic accuracy
Xian et al., 2022 [5]	CIP2A (P90)	Transistor-based (FET) biosensor module	HeLa cell lysate (validation reported); saliva-based use describe	Detection sensitivity: 1 × 10^−15^ g/mL	NR	NR	NR	NR
Chaibun et al., 2022 [21]	HPV-16 and HPV-18 genotypes	Electrochemical DNA genosensor (multianalyte)	Oral and cervical clinical samples	LOD: 22 fM (HPV-16); 20 fM (HPV-18)	NR	NR	NR	NR
Bhardwaj et al., 2025 [23]	IL-8	Electrochemical immunosensor (nano-ceria integrated microfluidic chip)	Saliva samples	LOD: 0.0001 ng/mL	NR	NR	NR	NR
Verzino et al., 2024 [17]	Neu5Ac (synthetic neuraminic acid sensor)	Whole-cell biosensor (chimeragenesis platform)	NR (platform development)	NR	NR	NR	NR	NR
Rashidova et al., 2024 [24]	IL-8	Optical fiber biosensor (label-free)	Saliva (protein detection described)	LOD: 0.91 fM	NR	NR	NR	NR
Wan et al, 2024 [13]	HER2 and CA15-3	Saliva-based biosensor (nanomaterial platform)	Saliva	Detection limit: as low as 1 fg/mL	NR	NR	NR	NR
Vetrivel et al., 2023 [25]	IL-8	Electrochemical immunosensor (MoS2-ZnO nanocomposite)	Saliva	LOD: 11.6 fM	Dynamic range: 500–400 pg/mL	NR	NR	NR
Jafari et al., 2020 [6]	Cyfra 21.1	Gold electrode-based immunosensor	Unprocessed human saliva	LLOQ: 2.5 ng/mL (LODNR)	Linear range: 2.5–50 ng/mL	NR	NR	NR
Wan et al., 2024 [4, 13]	P90 (CIP2A)	Field-effect transistor (FET)-based biosensor on disposable strips	Saliva and tissue	LOD: ~ 5 orders lower than commercial ELISA kits (absolute value NR)	NR	NR	NR	NR

### Detection of Interleukin-8 (IL-8)

Four studies centered on IL-8, a cytokine recognized for its role in tumorigenesis and immune modulation in OSCC. Vetrivel et al. developed a MoS₂-ZnO nanocomposite-based immunosensor, reporting a detection limit of 11.6 fM within the dynamic range of 500–4500 pg/mL [25]. Bhardwaj et al. employed a nano-ceria-integrated microfluidic electrochemical immunosensor, achieving an ultralow detection limit of 0.0001 ng/mL and quantifying IL-8 in saliva samples, with strong recovery values [23]. Similarly, Rashidova et al. demonstrated label-free IL-8 detection using a functionalized optical fiber biosensor with a limit of detection of 0.91 fM. Collectively, these platforms highlight IL-8 as a promising salivary biomarker and demonstrate that high analytical sensitivity can be achieved in saliva [24]. However, clinical diagnostic performance (sensitivity/specificity) and robustness to common oral confounders were not consistently reported, underscoring the need for standardized clinical validation.

### Detection of cytokeratin fragment 21.1 (Cyfra 21.1)

Jafari and Hasanzadeh introduced a gold electrode-based immunosensor capable of detecting Cyfra 21.1 directly in unprocessed saliva samples. The sensor demonstrated reliable performance across a linear range of 2.5–50 ng/mL, with a lower limit of quantification (LLOQ) of 2.5 ng/mL. This work highlights Cyfra 21.1 as a viable target for early OSCC diagnosis and illustrates the value of validating biosensors in minimally processed clinical matrices, where matrix effects are most relevant for point-of-care translation [23].

### Detection of P90 (CIP2A) in Oral Leukoplakia and OSCC

Two studies targeted the oncoprotein P90, also known as CIP2A, involved in the malignant transformation of oral leukoplakia. Wan et al. utilized a field-effect transistor (FET)-based biosensor, functionalized with anti-P90 antibodies on disposable glucose strips, detecting CIP2A in both saliva and tissue with a detection limit nearly five orders of magnitude lower than conventional ELISA kits [4]. A similar modular FET biosensor platform was developed by Xian et al., demonstrating a detection sensitivity of 1× 10⁻^15^ g/mL and validation using HeLa cell lysate [4]. These transistor-based biosensors offer advantages in miniaturization and rapid signal transduction. Nonetheless, clinical implementation will require clear reporting of calibration procedures, device stability, and performance in unprocessed saliva across diverse patient cohorts.

### HPV genotyping using electrochemical genosensors

Chaibun et al. presented a multianalyte electrochemical DNA biosensor targeting high-risk HPV genotypes 16 and 18, which are strongly associated with both cervical and oropharyngeal cancers. The device achieved detection limits of 22 fM for HPV-16 and 20 fM for HPV-18 and was validated in both oral and cervical clinical samples. The integration of sandwich hybridization with silica-redox dye and capture probes supported high analytical selectivity 21. To strengthen clinical relevance, future work should report diagnostic accuracy metrics (with confidence intervals) and clearly describe cohort composition, reference standards, and blinding procedures.

Pre-analytical variables are critical for salivary biomarker measurement and may contribute substantial variability across studies. Saliva collection protocol (stimulated vs unstimulated), time of day, patient preparation (fasting status, oral hygiene), sample processing (centrifugation/filtration), storage temperature and duration, and use of protease or enzyme inhibitors can all influence measured biomarker concentrations. In addition, common oral conditions and external exposures-including inflammation, periodontal disease, tobacco use, and recent food intake-should be considered as potential confounders when interpreting biomarker levels and evaluating diagnostic reliability.

Discussion of real-world applicability should extend beyond analytical performance. Key translational considerations include device stability and shelf-life, calibration and quality-control requirements, manufacturability and scalability of nanomaterials and microfabrication steps, cost per test, regulatory pathways, and end-user workflow in clinical or community screening settings. Implementation-focused comparisons across platforms (electrochemical, optical, and transistor-based) may help prioritize technologies that balance sensitivity with robustness, simplicity, and affordability.

### Critical comparison of biosensor performance

A comparative analysis of the included studies reveals that transistor-based biosensors (e.g., for CIP2A detection) generally offer the highest sensitivity, reaching femtomolar (fM) or even attomolar ranges, outperforming traditional electrochemical immunosensors. However, electrochemical platforms demonstrate superior robustness when handling unpurified saliva samples due to their compatibility with microfluidic integration. While optical fiber sensors provide excellent label-free detection, their requirement for precise alignment and specialized light sources currently limits their portability compared to electrochemical handheld devices. The choice of nanomaterials, such as nano-ceria or MoS2-ZnO, significantly enhances the signal-to-noise ratio, yet the variability in LOD across studies suggests a lack of standardized testing protocols in the field.

### Translational challenges and clinical validation

Despite the promising analytical performance of these biosensors, significant hurdles remain for clinical translation. First, the majority of the reviewed studies utilized small sample cohorts, which limits the statistical power required for clinical diagnostic approval. Second, the "matrix effect" of whole saliva, which contains a complex mixture of proteins and enzymes, remains a challenge for sensors that have only been validated in buffered solutions. For these technologies to transition from the laboratory to the clinic, future research must prioritize large-scale longitudinal clinical trials and the development of fully integrated, user-friendly devices that require no sample pre-treatment.

## Conclusion

Biosensors represent a promising advancement in oral cancer diagnostics by enabling non-invasive and potentially cost-effective detection of disease-associated biomarkers. Across the included studies, multiple platforms achieved very low analytical detection limits for targets such as IL-8, Cyfra 21.1, CIP2A (P90), and high-risk HPV genotypes. However, the current evidence base is dominated by early-stage analytical and prototype investigations, and clinically validated diagnostic performance (sensitivity/specificity against appropriate reference standards) is incompletely reported. Therefore, before biosensors can be adopted for routine screening, future research should emphasize standardized analytical validation (including matrix effects and pre-analytical saliva variables), reproducibility testing, and well-designed multicenter clinical studies. Practical implementation issues—such as device stability, calibration, manufacturing scalability, regulatory pathways, and cost—should also be addressed, alongside the development of multiplexed platforms for simultaneous biomarker detection.

## Data Availability

No datasets were generated or analysed during the current study.
